# Sea Buckthorn Pomace Supplementation in the Diet of Growing Pigs—Effects on Fatty Acid Metabolism, HPA Activity and Immune Status

**DOI:** 10.3390/ijms19020596

**Published:** 2018-02-21

**Authors:** Dirk Dannenberger, Margret Tuchscherer, Gerd Nürnberg, Marion Schmicke, Ellen Kanitz

**Affiliations:** 1 Leibniz Institute for Farm Animal Biology, Institutes of Muscle Biology and Growth, Behavioural Physiology, and Genetics and Biometry, Wilhelm-Stahl-Allee 2, 18196 Dummerstorf, Germany; mtuchsch@fbn-dummerstorf.de (M.T.); gnuernbg@fbn-dummerstorf.de (G.N.); ellen.kanitz@fbn-dummerstorf.de (E.K.); 2 Clinic for Cattle Endocrinology Laboratory, University of Veterinary Medicine Hannover, Bischofsholer Damm 15, 30173 Hannover, Germany; marion.schmicke@tiho-hannover.de

**Keywords:** pig, sea buckthorn pomace, *n*-3 PUFA, mRNA expression, hypothalamus, immunity

## Abstract

There is evidence that sea buckthorn, as a source of *n*-3 polyunsaturated fatty acids (*n*-3 PUFA), possesses health-enhancing properties and may modulate neuroendocrine and immune functions. In the present study, we investigated the effect of sea buckthorn pomace (SBP) supplementation in the diet of growing German Landrace pigs on fatty acids in the blood and hypothalamus, peripheral immune parameters and mRNA expression of corticotropin-releasing hormone (CRH), mineralocorticoid receptor (MR) and glucocorticoid receptor (GR) in the hypothalamus and spleen. Pigs were fed diets supplemented with 12% of dried SBP or 0% SBP (control group) over an intervention period of eight weeks. The fatty acid profiles in blood plasma were significantly affected by SBP supplementation only for C18:2*n*-6 and *n*-6/*n*-3 PUFA ratio compared with the control group. SBP supplementation did not significantly affect the fatty acid concentrations in the hypothalamus. Furthermore, there were no significant differences in mRNA expression of CRH, MR and GR in the hypothalamus or of GR mRNA expression in the spleen. Concerning the immune status, the plasma IgG levels tended to be higher in SBP pigs, whereas the leukocyte distribution, mitogen-stimulated lymphocyte proliferation, and serum IgM levels remained unchanged. In conclusion, the SBP supplementation of the diet only caused moderate effects on fatty acid metabolism, but no significant effects on hypothalamic–pituitary–adrenal (HPA) activity and immunity in growing pigs. It seems that a beneficial effect of dietary *n*-3 PUFA on health and welfare is more likely to be expected during stressful situations.

## 1. Introduction

There is ample evidence that sea buckthorn (*Hippophae rhamnoides* L.), as a source of *n*-3 polyunsaturated acids (*n*-3 PUFA), possesses health enhancing and therapeutic properties, and it may modulate neuroendocrine and immune functions. In addition, lipids of sea buckthorn have been proven an excellent source of other essential fatty acids, such as linoleic (18:2*n*-6) and oleic acid (18:1*cis*-9), and contain β-carotene, vitamin E and secondary plant metabolites like flavonoids, which function as chain-breaking antioxidants. Research in rats has shown that the administration of sea buckthorn extract improves endocrine and metabolic status that was disturbed by immobilization stress [1]. Furthermore, it is well known that sea buckthorn extract may protect against infections, eliminate inflammation and support the treatment of many diseases [2,3,4]. Additionally, studies have demonstrated the efficacy of sea buckthorn seed oil in reducing dyslipidemia, cardiovascular risk factors and hypertension in humans [5]. The lipid fraction of sea buckthorn contains on average 6–13% α-linolenic acid (18:3*n*-3) and has a low ratio of *n*-6/*n*-3 polyunsaturated fatty acids (PUFA) of 1.1–1.3. Long-chain *n*-3 PUFA, mainly docosahexaenoic acid (22:6*n*-3, DHA), is the most abundant *n*-3 PUFA in the central nervous system of humans and is suggested to play an essential role in brain development and function [6]. The recognition that *n*-3 PUFAs exhibit stress-regulating and anti-inflammatory properties in human and animal models has prompted studies investigating their efficacy for growth, immunity and regulation of the hypothalamic–pituitary–adrenal (HPA) axis. Thus, it was shown that fatty acids may alter the feedback of the HPA axis via corticotrophin releasing hormone (CRH) secretion and glucocorticoid receptor functioning [7,8]. Fatty acids are also known to play an important role in immune cell regulation, for instance, as a source of energy and as structural components of cell membranes with influences on immune cell activation and functional responses [9].

Despite the unique nutritional properties of sea buckthorn, information on its efficacy for growth, immunity and regulation of the HPA axis is sparsely described in existing literature. Recently, the effects of dried sea buckthorn pomace (SBP) supplementation on growth characteristics, meat quality and fatty acid profiling of longissimus muscle in finishing Landrace pigs were described [10]. Here, SBP supplementation (concentrations of 0%, 4%, 8% and 12%; intervention duration of 4 or 8 weeks) was assessed in the muscle of German Landrace pigs. In the present study, pigs of two diet groups with maximally different diet composition—a control group (CG) and a feeding group (12% SBP over 8 weeks)—were selected for investigations of immune status, mRNA expression and fatty acid profile. We hypothesize that SBP supplementation, with lipids high in linolenic acid (18:3*n*-3), lead to an increase of precursor and de novo synthesized long-chain *n*-3 PUFAs in the hypothalamus combined with positive effects on immune responses in pigs. The focus of the present study was the investigation of fatty acid concentrations and mRNA expression of HPA-regulating hormones and receptors in the hypothalamus and peripheral immune responses as well as fatty acid profiles in blood plasma of SBP supplement-fed vs. control-fed pigs.

## 2. Results

### 2.1. Fatty Acid Profiling

The fatty acid profiling in blood plasma of Landrace pigs fed control (CG) vs. sea buckthorn pomace (SBP) supplemented (FG-SBP) diet before and after the SBP intervention is shown in Table 1. Before the SBP intervention, there were no significant differences in any fatty acid values observed between CG and FG-SBP pigs. There was an overall effect of SBP supplementation vs. control on single saturated fatty acids (SFA) C14:0, C15:0, and sum polyunsaturated fatty acids (PUFA). Further, the multiple pairwise comparisons by Tukey–Kramer revealed that SBP supplemented pigs displayed higher C18:2*n*-6 proportion and *n*-6/*n*-3 PUFA ratio in blood plasma compared with the control group. The highest fatty acid proportions were measured for 18:2*n*-6, 18:1*cis*-9 and 16:0 in both diet groups. In addition, the results showed significant differences in the time points TP0 and TP1 for single und sum SFA, mono unsaturated fatty acids (MUFA) and PUFA proportions in both diet groups (Table 1).

### 2.2. Biochemical Parameters in Blood Plasma

The biochemical parameters in blood plasma showed only moderate differences between the two diet groups (Table 2). There was an overall effect of SBP intervention on plasma glucose, cortisol concentration and IgG level. However, the Tukey–Kramer analyses revealed significant differences between the CG- and FG-SBP group for these parameters before the SBP intervention. For glucose, there was an increase in the CG group and no significant changes in the FG-SBP group from TP0 to TP1. The cortisol concentrations showed lower levels after the treatment period (TP1) in the CG group, whereas no significant effect was found on cortisol in the SBP group (Table 2). In addition, the IgG concentrations in plasma of SBP-supplemented pigs were significantly higher both before and after SBP intervention compared to CG animals. Indeed, the SBP pigs displayed in tendency a higher IgG concentration at TP1 compared to TP0 (*p* = 0.09). The concentrations of triglycerides, cholesterol, free fatty acids, lactate, protein and IgM were not affected by SBP intervention, but there was an overall effect of time for triglycerides and protein concentrations. The Tukey–Kramer test showed an increase in protein levels for both groups from TP0 to TP1.

### 2.3. Immune Parameters in Peripheral Blood

The immune parameters in blood plasma of Landrace pigs fed control (CG) vs. sea buckthorn pomace supplemented (FG-SBP) diet are shown in Table 3. There was no significant effect of SBP intervention on the number of peripheral blood mononuclear cells (PBMCs); the proliferation/viability of PBMCs in response to ConA and LPS; the percentages of lymphocytes, monocytes and neutrophils; or on the neutrophil to lymphocyte ratio.

### 2.4. Fatty Acid Concentration in Hypothalamus Tissue

The fatty acid concentrations in hypothalamus tissue were not affected by SBP intervention (Table 4). Neither single/sum SFA nor MUFA nor PUFA concentrations in hypothalamus tissue showed significant differences between the two diet groups.

### 2.5. Hypothalamus mRNA Expression Levels

**Figure 1:**
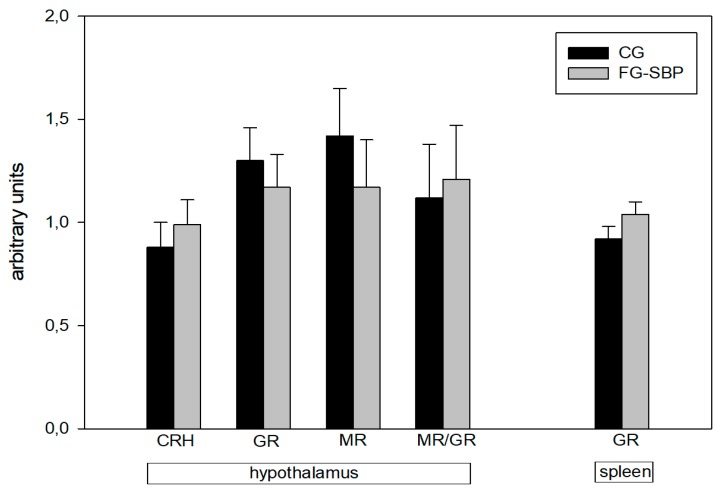
The mRNA expression levels (arbitrary units) of selected genes in the hypothalamus and spleen of Landrace pigs fed control (CG) vs. sea buckthorn pomace supplemented (FG-SBP) diet. CRH: corticotropin-releasing hormone, GR: glucocorticoid receptor, MR: mineralocorticoid receptor, MR/GR: mineralocorticoid receptor/glucocorticoid receptor ratio.

In addition, mRNA expression levels of corticotropin-releasing hormone (CRH), mineralocorticoid receptor (MR), glucocorticoid receptor (GR) and the MR/GR ratio in the hypothalamus, as well as the GR mRNA in the spleen, were not affected by diet (Figure 1).

## 3. Discussion

Generally, effective functioning of the immune system is required for protection against a number of diseases, which can undesirably affect the performance and welfare of farm animals. It has been shown in pigs that dietary *n*-3 PUFA supplements, mainly linseed, rapeseed oils/meals and fish oil, may beneficially reduce inflammatory immune reactions and improve indices of specific immune responses [11,12,13,14]. However, the efficacy of sea buckthorn on the endocrine and immune status in pigs has not been described so far. The present study examined the hypothesis that sea buckthorn pomace (SBP) supplementation would lead to an increase of precursor and de novo synthesized long-chain *n*-3 PUFAs in the hypothalamus combined with positive effects on immune responses in pigs. However, according to our results, we cannot confirm our hypothesis of an increase of precursor and de novo synthesized long-chain *n*-3 PUFAs in the hypothalamus combined with positive effects on immune responses in SBP supplementation (12% over duration of 8 weeks) to German Landrace castrates. The single and sum of *n*-3 PUFAs in blood plasma were not significantly affected by SBP supplementation. However, the SBP intervention caused a higher C18:2*n*-6 proportion and the corresponding *n*-6/*n*-3 PUFA ratio in blood plasma compared with the control pigs. Since alterations of fatty acids in blood plasma reflect more short-term effects of diets, the present findings could be attributed to differences in fatty acid composition with respect to C18:2*n*-6 of the diets on the day of sampling. The main reason for the unchanged plasma fatty acid proportions with respect to *n*-3 PUFA could be the low differences in the PUFA proportions of the two diets. Although there was a lower *n*-6/*n*-3 PUFA ratio in the diet of the FG-SBP group compared with the CG group (6.7 vs. 8.1), the analyzed *n*-3 PUFA proportions in the diet of the FG-SBP group were not much higher compared with the CG group (Table 5). It seems that the *n*-3 PUFA content in the SBP-supplemented diet was too low to provoke meaningful impact on fatty acid metabolism. Also, the fatty acid concentrations in the hypothalamus tissue of pigs were not diet affected, resulting in unchanged values for fatty acid as well as for SFAs, MUFAs and *n*-3/*n*-6 PUFAs. One reason could be the different PUFA responsiveness of various cerebral brain lobes. A study with juvenile pigs fed a fish oil supplemented diet revealed that *n*-3 PUFA intervention resulted in different responses of the frontal, parietal, occipital and temporal brain lobes. The hypothalamus, as part of the temporal lobe, appeared to be less responsive to dietary *n*-3 PUFA intervention compared with the other brain lobes [15]. Dietary 18:3*n*-3 has been shown to be desaturated and elongated to 22:6*n*-3 (DHA) and finally incorporated in predominantly neural tissues, even in the absence of dietary DHA [15]. This is in agreement with the results of the present study, which detected DHA concentrations in the hypothalamus tissue of both diet groups that were approximately 20 times higher compared with the corresponding 18:3*n*-3 concentrations but not diet affected. Hence, the biochemical parameters in pig blood plasma showed moderate diet-affected variations for glucose, cortisol and IgG. However, the evaluation of these results is limited by the fact that there are baseline differences between the feeding groups before the SBP intervention. Although the SBP intervention did not significantly affect the concentrations of glucose and cortisol, there was a numeric decrease of these parameters in the FG-SBP group from TP0 to TP1. Several studies in humans and animals have shown that sea buckthorn extract may modulate glucose metabolism with hypoglycemic functions [16,17]. Further, supplementation with sea buckthorn could suppress the levels of cortisol and ACTH in rats with chronic stress and reduce plasma cortisol concentrations following cold stress in humans, indicating a higher stress tolerance capacity and adaptation [18,19]. Relating to IgG, the concentrations were significantly elevated in the plasma of SBP-supplemented pigs compared to the values in the CG group as well as before and after SBP intervention. In addition, in SBP pigs, we found in tendency a higher IgG concentration at the end of the intervention than at the beginning. Similarly, a linseed oil supplement fed to finishing pigs also resulted in increased plasma IgG concentrations in the pigs [20]. Although most studies have investigated the effects of dietary *n*-3 PUFA on immune responses and growth in suckling and weanling piglets [21,22,23], immunomodulatory effects of *n*-3 PUFA have also been observed in growing/finishing pigs [11]. It is well known that blood leukocyte composition and lymphocyte proliferation in pigs are influenced by a variety of environmental factors, including diet, age and housing conditions [24,25]. In the present study, the leukocyte distribution and mitogen-stimulated lymphocyte proliferation did not differ between pigs fed the two diets. These findings are in accordance with reports in weanling pigs fed an *n*-3 PUFA-enriched diet [26]. However, studies in finishing pigs have shown that diets supplemented with 12% camelina oil cakes, lipids rich in 18:3*n*-3, or an *n*-6/*n*-3 PUFA ratio of 1:1 can alleviate the protein and gene expression of pro-inflammatory cytokines in the spleen, skeletal muscle and adipose tissue [13,27]. Thus, these studies demonstrate the anti-inflammatory properties of *n*-3 PUFAs. However, the growth performance of the pigs was not affected. Additionally, that is in accordance with the results of the present study, showing unaffected average daily gain (ADG) during the 8 weeks of 12% SBP supplementation in Landrace pigs [10]. It seems that under optimal circumstances, the animals reach their genetic potential, making it difficult to observe any beneficial effects of dietary *n*-3 PUFA on growth performance and immune responses. However, during stressful conditions, such as weaning and acute or chronic inflammatory states, dietary SBP supplementation as an important source of *n*-3 PUFA and polyphenolic antioxidants may beneficially modulate the immune system of pigs, which can positively influence the health and welfare of the animals.

## 4. Materials and Methods

### 4.1. Animal Study

Materials and methods in this study, 58 German Landrace castrates (from two half-sib groups) were kept at the experimental station of the Leibniz Institute for Farm Animal Biology in Dummerstorf. Animal keeping and feeding were in accordance with the animal care regulations (No. 7221.3-2.1-019/13). The pigs were randomly assigned into six feeding groups at a mean life weight of 28 kg (9 to 10 pigs per pen). The experimental design of the pig intervention study was recently described in detail [10]. Pigs were fed diets supplemented with different concentrations of dried sea buckthorn pomace (SBP) over different intervention durations. The pigs with the most different diet composition—control group (CG) and sea buckthorn pomace supplemented group (FG-SBP, 12% SBP over 8 weeks)—were selected for fatty acid metabolism, HPA activity and immune status investigations. The composition of the diets is shown in Table 5. Blood samples of pigs of both diet groups were taken before (113th day of life) and after SBP supplementation (162nd day of life). The blood samples were taken in EDTA-containing tubes for various analyses. Pigs were slaughtered at the abattoir of the Leibniz Institute for Farm Animal Biology in Dummerstorf (Germany) within two weeks according to the EU regulations. Immediately after slaughtering, brains and spleens were quickly removed (<5 min). The hypothalamus was dissected out of both brain hemispheres, and all tissue samples were snap-frozen in liquid nitrogen and stored at −80 °C until lipid and gene expression analyses.

### 4.2. Sample Preparation for Fatty Acid Analysis—Blood Plasma

EDTA blood samples were centrifuged at 2000× *g* for 15 min at 4 °C to separate plasma for the following fatty acid analysis. Plasma samples, approximately 1.5 g, were dropwise added to 8 mL of chloroform/methanol (2:1, *v*/*v*) at room temperature. The solution contained C19:0 as an internal standard. The detailed sample preparation procedure has been recently described [28]. Briefly, all the solvents contained 0.005% (*w*/*v*) of *t*-butylhydroxytoluene (BHT) to prevent the oxidation of PUFAs. The extraction mixtures were stirred two times for 15 min and stored at 5 °C for 18 h in the dark and subsequently washed with 0.02% CaCl_2_ solution. After centrifugation (2500 rpm, 5 min), the organic phase was dried with Na_2_SO_4_ and K_2_CO_3_ (10:1, *w*/*w*), and the solvent was subsequently removed under gentle nitrogen stream at room temperature. The total lipid contents were stored at −18 °C until transmethylation of fatty acids. Sodium methoxide in methanol was added to the extracts, which were shaken in a 60 °C water bath for 10 min. Subsequently, 1 mL of 14% boron trifluoride (BF_3_) in methanol was added to the mixture, which was then shaken for an additional 10 min at 60 °C. Finally, the fatty acid methyl esters (FAME) were resuspended in 500 µL of *n*-heptane and stored at −18 °C until use for gas chromatography (GC) analysis.

### 4.3. Sample Preparation for Fatty Acid Analysis—Hypothalamus

Total lipid samples of approximately 0.3 grams were extracted in duplicate with chloroform/methanol (2:1, *v*/*v*) by using an Ultra Turrax homogenizer (3 × 15 s, 12,000 U/min; IKA Werke, Staufen, Germany) at room temperature, as recently described in detail [28]. Briefly, the final lipid extracts were redissolved in 300 µL of toluene, and an aliquot was used for methyl ester preparation. Sodium methoxide in methanol was added to the samples, which were shaken in a 60 °C water bath for 10 min. Subsequently, 1 mL of 14% boron trifluoride (BF_3_) in methanol was added to the mixture, which was then shaken for an additional 10 min at 60 °C. Finally, the FAMEs were resuspended in 100 µL of *n*-hexane and stored at −18 °C until use for gas chromatography (GC) analysis.

### 4.4. Analysis of Fatty Acids

The fatty acid analysis was performed using capillary GC with a CP-Sil 88 CB column (100 m × 0.25 mm, Agilent, Santa Clara, CA, USA) that was installed in a PerkinElmer gas chromatograph CLARUS 680 with a flame ionization detector and split injection (PerkinElmer Instruments, Shelton, CT, USA). The detailed GC conditions were recently described [29]. Briefly, the initial oven temperature was 150 °C, which was held for 5 min; subsequently, the temperature was increased to 175 °C and then to 200 °C at a rate of 2 °C min^−1^ and held for 10 min. Finally, the temperature was increased to 225 °C at a rate of 1.5 °C min^−1^ and held for 25 min. Hydrogen was used as the carrier gas at a flow rate of 1 mL min^−1^. The split ratio was 1:20, and the injector and detector were set at 260 and 280 °C, respectively. The quantification of fatty acids was done by the use of C19:0 as the internal standard. For the calibration procedure, the reference standard mixture “Sigma FAME” (Sigma-Aldrich, Deisenhofen, Germany), the methyl ester of C18:1*cis*-11, C22:5*n*-3, C18:2*cis*-9, *trans*-11 (Matreya, State College, PA, USA), C22:4*n*-6 (Sigma-Aldrich, Deisenhofen, Germany) and C18:4*n*-3 (Larodan, Limhamn, Sweden) were used. The five-point calibration of single fatty acids ranged between 16 and 415 mg/mL and was checked after GC analysis of five samples.

### 4.5. Biochemical Analyses

EDTA blood samples were centrifuged at 2000× *g* for 15 min at 4 °C to separate plasma for the following biochemical analyses. Triglycerides, cholesterol, glucose and free fatty acids were measured in plasma samples using an automatic clinical chemistry analyzer (ABX PENTRA 400, HORIBA ABX International, AxonLAB, Reichenbach, Germany). For triglycerides, a test kit, Triglycerides CPO ABX pentra A11AO1640, was used. The intra-assay coefficient of variation (CV) was 1.51%. For cholesterin in plasma samples, the CHOD-PAP Liquicolor cholesterin test kit (mti diagnostcs, Idstein, Germany) was used. The intra-assay CV was 2.43%. For glucose determination, the Glucose Hexokinase Fluid 5+1 Serum test kit (mti diagnostics, Idstein, Germany) was used. The intra-assay CV was 3.08%. The free fatty acids were analyzed using the NEFA C ACS-ACOD test kit method (Wako Chemicals, Neuss, Germany). The intra-assay CV was 5.73% [30]. Plasma lactate was determined by an enzymatic-spectrophotometric assay (Labor+Technik Eberhard Lehmann, Berlin, Germany). The sensitivity of the test was 0.11 mmol/L, and the intra- and inter-assay CVs were <2.9%. Plasma cortisol concentrations were analyzed in duplicate using a commercially available ELISA kit (DRG Instruments, Marburg, Germany) according to the manufacturer’s guidelines. The assay was validated for use with porcine plasma. The test sensitivity was 3.4 ng/mL, and the intra- and inter-assay CV values were 6.1% and 9.1%, respectively. Total protein content was determined by the biuret method (Bioquant^®^ Protein 110307; Merck, Darmstadt, Germany). Concentrations of immunoglobulins IgG and IgM were determined in duplicate with a porcine-specific ELISA according to the manufacturer’s instructions (Bethyl, Laboratories Inc., Montgomery, TX, USA). The intra-assay and inter-assay CVs for these analyses were <5% and <10%, respectively.

### 4.6. RNA Isolation and Quantification of Transcripts

Total RNA was isolated from hypothalamus and spleen samples with the RNeasy Lipid Tissue Kit (Qiagen, Hilden, Germany) as recommended by the supplier. The RNA was quantified in a NanoPhotometer™ (IMPLEN, München, Germany). The quality of RNA was monitored with the Experion Automated Electophoresis System (BIO-RAD, München, Germany) according to the manufacturer’s protocol. All samples were classified in the acceptable quality category as determined by an RNA quality indicator >8.5. The mRNA expression of the *NR3C1* gene encoding the glucocorticoid receptor (GR), of the *NR3C2* gene encoding the mineralocorticoid receptor (MR) and of the *CRH* gene was monitored using reverse transcription (RT) with subsequent real-time polymerase chain reaction (PCR) as described previously [31]. Reverse transcription was carried out with 500 ng of total RNA using an iScript cDNA synthesis kit (BIO-RAD, München, Germany) following the guidelines of the manufacturer. The resulting cDNA was amplified by real-time PCR (iCycler, BIO-RAD, München, Germany) using an iQ SYBR Green Supermix (BIO-RAD, München, Germany). One microliter of the RT reaction solution was added to 10 µL of PCR mix primed with gene-specific oligonucleotides (TIB MOLBIOL, Berlin, Germany). Based on the published cDNA and gene sequences (GR: accession no. AY779185; MR: accession no. M36074; CRH: accession no. NM_001113062), the primers were designed to span a corresponding intron and to anneal between 60 and 70 °C. The following primer sequences were used: GR (forward, 5´-GTTCCAGAGAACCCCAAGAGTTCA-3´; reverse, 5´-TCAAAGGTGCTTTGGTCTGTGGTA-3´), MR (forward, 5´-GTCTTCAAAAGAGCCGTGGAA-3´; reverse, 5´-CTCCTCGTGGAGGCCTTTTAACTT-3´), CRH (forward, 5´-CTCAGAGCCCAAGTCCGTTGAGAGA-3´; reverse, 5´-GCATTTTAGGGGCGCTAGCTTCTGA-3´). PCR was carried out using a hot start (3 min, 95 °C; 30 s, 60 °C; 45 s, 70 °C), 39 additional cycles (10 s, 95 °C; 30 s, 60 °C; 45 s, 70° C) and with a final cycle of 10 s, 95 °C; 30 s, 60 °C; 7 min, 70 °C, corresponding to denaturation, annealing and elongation, respectively. The specificity of the products was assessed using a melting point analysis that started at 60 °C and elevated to 90 °C (1 °C per 10 s) as well as by agarose gel electrophoresis (2%). The oligonucleotide structure was verified by sequencing in a subset of the experiments. The relative quantification was performed using the quantification module in CFX Manager Software™ version 2.1 (BIO-RAD, München, Germany) based on the PCR efficiency and crossing point deviation of an unknown sample versus a control and standardization by non-regulated reference genes [32,33]. Data for mRNA expression of genes investigated are presented as relative expression ratio normalized to *ACTB* and *TBP* as reference genes. The ratio of MR/GR mRNA was computed.

### 4.7. Immune Parameter Analysis

The mitogens concanavalin A (6.25 µg/mL; ConA) and lipopolysaccharide (12.5 µg/mL; LPS) were used in a lymphocyte proliferation/viability assay as previously described [34]. Peripheral blood mononuclear cells (PBMCs) were isolated from heparinized blood by density gradient centrifugation, and the cell concentration was adjusted to 2 × 10^6^ cells/mL in complete RPMI 1640 medium. Cell suspensions were added in triplicate to flat-bottom 96-well plates at a volume of 200 µL per well, and the plates were incubated in a 5% CO_2_-humidified incubator at 37 °C for 72 h. Cell proliferation/viability was evaluated using a 3-[4,5-dimethylthiazol-2-yl]-2,5 diphenyl tetrazolium bromide assay (Roche Diagnostics, Mannheim, Germany). The optical density (OD) was measured using a microplate reader (Dynatech, Denkendorf, Germany) with a test wavelength of 550 nm and a reference wavelength of 690 nm. The results were expressed as a mitogen-stimulated proliferation index (PI), which was calculated as the ratio of the OD in the presence of mitogen to the OD in the absence of mitogen.

Relative leukocyte counts (lymphocytes, monocytes and neutrophils) were made by microscopic examination of the smears stained using the May–Grünwald–Giemsa procedure. Neutrophil to lymphocyte (N/L) ratios were determined from counts of at least 200 leukocytes in total.

### 4.8. Statistical Analysis

The statistical analyses were performed by using the SAS© program package (9.4, SAS Institute, Cary, NC, USA, 2012) for two-way ANOVA with factor “Feeding SBP” (0 vs. 12%) and repeated factor “intervention time” (0 vs. 8 weeks). The covariance structure of the repeated factor was modeled as unstructured. In the tables, least squares means (LSMs) and standard error mean (SEM) of the investigated parameters are presented. The results were considered statistically significant at *p* ≤ 0.05. For multiple comparison adjustment, we used the Tukey–Kramer correction.

## 5. Conclusions

In conclusion, the SBP supplementation of the diet caused only moderate effects on fatty acid metabolism and immunity in Landrace pigs. It seems that the *n*-3 PUFA content in the SBP supplemented diet was too insufficient and/or the intervention period was too short to provoke a stronger impact on neuroendocrine modulated immune functions in growing pigs.

## Figures and Tables

**Table 1:** Fatty acids in blood plasma (% of total fatty acids) of Landrace pigs fed control (CG) vs. sea buckthorn pomace supplemented (FG-SBP) diet before and after sea buckthorn pomace (SBP) intervention.

	CG	FG-SBP	CG	FG-SBP	*p*-Value	*p*-Value	*p*-Value
	Timepoint 0 (TP0) ^§^		Timepoint 1 (TP1) ^§^		CG vs. FG-SBP	TP 0 vs. TP 1	SBP * TP
	LSM_SEM_ (*n* = 10)	LSM_SEM_ (*n* = 10)	LSM_SEM_ (*n* = 10)	LSM_SEM_ (*n* = 10)			
			
SFA							
C12:0	0.59_0.08_^a^	0.64_0.08_^a^	0.09_0.01_^b^	0.09_0.01_^b^	0.7680	<0.0001	0.6479
C14:0	0.98_0.03_^a^	0.93_0.03_^a,b^	0.65_0.03_^b^	0.58_0.03_^b^	0.0297	<0.0001	0.8261
C15:0	0.46_0.05_^a^	0.33_0.05_^a^	0.36_0.02_^a,b^	0.28_0.02_^b^	0.0162	0.0786	0.5120
C16:0	15.72_0.32_	15.33_0.32_	16.22_0.34_	15.76_0.34_	0.2908	0.0867	0.8897
C18:0	10.70_0.20_^a^	11.15_0.20_^a^	11.98_0.17_^b^	11.92_0.17_^b^	0.2652	<0.0001	0.2202
Sum SFA*	39.86_0.79_^a^	38.44_0.79_^a^	34.30_0.40_^b^	33.83_0.40_^b^	0.1261	<0.0001	0.4788
MUFA							
C16:1*cis*-9	1.26_0.05_^a^	1.26_0.05_^a^	1.63_0.10_^b^	1.60_0.10_^b^	0.8417	0.0016	0.8493
C18:1*cis*-9	16.38_0.45_^a^	16.41_0.45_^a^	18.81_0.49_^b^	17.87_0.49_^b^	0.2557	0.0021	0.3828
C18:1*cis*-11	1.41_0.05_^a^	1.37_0.05_^a^	1.72_0.08_^b^	1.72_0.08_^b^	0.7300	0.0001	0.7724
Sum MUFA ^#^	20.86_0.63_^a^	20.33_0.63_^a^	23.88_0.52_^b^	22.58_0.52_^b^	0.0689	0.0009	0.5690
PUFA							
C18:2*n*-6	25.02_0.68_^a^	25.60_0.68_^a^	25.59_0.75_^a^	27.57_0.75_^b^	0.1226	0.0578	0.2790
C18:3*n*-3	0.72_0.04_^a^	0.71_0.04_^a^	0.93_0.09_^b^	0.99_0.08_^b^	0.7748	0.0003	0.4793
C20:4*n*-6	9.31_0.41_	10.27_0.41_	10.16_0.33_	9.86_0.33_	0.3996	0.5468	0.0912
C20:5*n*-3	0.56_0.03_^a^	0.63_0.03_^a^	0.87_0.04_^b^	0.80_0.04_^b^	0.9143	<0.0001	0.0934
C22:5*n*-3	1.26_0.05_^a^	1.37_0.05_^a^	1.64_0.05_^b^	1.53_0.05_^b^	0.9677	<0.0001	0.0470
C22:6*n*-3	0.92_0.09_^a^	0.98_0.09_^a^	0.71_0.04_^b^	0.60_0.04_^b^	0.7586	0.0009	0.2987
Sum PUFA ^+^	39.28_0.91_^a^	41.23_0.91_^a^	41.82_0.60_^b^	43.56_0.60_^b^	0.0225	0.0062	0.9129
*n*-6/*n*-3 PUFA ratio	10.30_0.30_^a^	10.15_0.30_^a^	9.07_0.30_^b^	10.10_0.30_^a^	0.2110	0.0281	0.0416

^§^ Timepoint (TP) 0—before SBP intervention, 113th day of life; ^§^ timepoint 1—after eight weeks of SBP intervention, 162nd day of life, SBP*TP—interaction of timepoint (TP) and sea buckthorn pomace (SBP), * Sum saturated fatty acids (SFA): 10:0 + 11:0 + 12:0 + 13:0 + 14:0 + 15:0 + 16:0 + 17:0 + 18:0 + 20:0 + 21:0 + 22:0 + 23:0 + 24:0; ^#^ Sum mono unsaturated fatty acids (MUFA): 14:1 + 15:1 + 16:1 + 17:1 + 18:1*trans* + 18:1*cis*-9 + 18:1*cis*-11 + 22:1 + 24:1; ^+^ Sum polyunsaturated fatty acids (PUFA): 18:2*trans* + 18:2*n*-6 + 18:3*n*-3 + 18:4*n*-3 + 20:3*n*-6 + 20:4*n*-6 + 20:5*n*-3 + 22:4*n*-6 + 22:5*n*-3 + 22:6*n*-3 + 18*cis*-9, *trans*-11CLA + 18:3*n*-6+ 20:2*n*-6 + 20:3*n*-3 + 22:2*n*-6; ^a,b^ significant between feeding groups (*p* ≤ 0.05). LSM, least squares means; SEM, standard error mean.

**Table 2:** Biochemical parameters in blood plasma of Landrace pigs fed control (CG) vs. sea buckthorn pomace supplemented (FG-SBP) diet before and after SBP intervention.

	CG	FG-SBP	CG	FG-SBP	*p*-Value	*p*-Value	*p*-Value
	Timepoint 0 (TP0) ^§^		Timepoint 1 (TP1) ^§^		CG vs. FG-SBP	TP 0 vs. TP 1	SBP * time point
	LSM_SEM_ (*n* = 10)	LSM_SEM_ (*n* = 10)	LSM_SEM_ (*n* = 10)	LSM_SEM_ (*n* = 10)			
			
Triglycerides (mmol/L)	0.42_0.05_	0.46_0.05_	0.33_0.03_	0.36_0.03_	0.4844	0.0298	0.8820
Cholesterol (mmol/L)	2.34_0.06_	2.31_0.06_	2.27_0.06_	2.34_0.06_	0.7855	0.7476	0.4222
Glucose (mmol/L)	3.83_0.20_^a^	5.51_0.20_^b^	4.43_0.22_^b^	4.83_0.22_^b^	0.0003	0.8110	0.0032
FFA (mmol/L)	57.61_9.01_	62.36_9.01_	108.6_26.65_	45.00_26.65_	0.1883	0.3648	0.0750
Lactate (mmol/L)	2.94_0.46_	2.15_0.46_	2.54_0.62_	2.35_0.62_	0.4217	0.8450	0.5513
Cortisol (ng/mL)	34.37_2.94_^a^	14.61_2.94_^b^	24.02_3.39_^c^	12.79_3.39_^b^	0.0007	0.0188	0.0870
Protein (mg/mL)	69.82_0.94_^a^	70.41_0.94_^a^	72.02_1.07_^b^	72.88_1.07_^b^	0.4979	0.0260	0.8926
IgG (mg/mL)	10.92_0.64_^a^	12.61_0.64_^b^	10.10_0.45_^a^	13.55_0.45_^b^	0.0005	0.9091	0.0925
IgM (mg/mL)	5.23_0.47_	5.51_0.47_	6.11_0.58_	5.57_0.58_	0.8461	0.1912	0.2464

^§^ Timepoint 0—before SBP intervention, 113th day of life; ^§^ timepoint 1—after eight weeks of SBP intervention, 162nd day of life; ^a,b,c^ significant between feeding groups (*p* ≤ 0.05); FFA—free fatty acids.

**Table 3:** Immune parameters in peripheral blood of Landrace pigs fed control (CG) vs. sea buckthorn pomace supplemented (FG-SBP) diet.

	CG	FG-SBP	*p*-Value (*p* < 0.05)
	LSM_SEM_ (*n* = 10)	LSM_SEM_ (*n* = 10)	
	
PBMC × 10^6^	8.31_0.67_	8.81_0.67_	0.6072
PI-ConA	3.36_0.20_	3.36_0.20_	0.9972
PI-LPS	2.08_0.11_	2.03_0.11_	0.7598
Lymphocytes (%)	45.76_2.33_	44.99_2.33_	0.8187
Monocytes (%)	8.14_0.56_	8.66_0.54_	0.5224
Neutrophils (%)	27.21_2.34_	30.83_2.36_	0.2911
Neutrophil to lymphocyte (N/L) ratio	0.62_0.08_	0.73_0.08_	0.3773

**Table 4:** Fatty acid concentrations (mg/g tissue) in hypothalamus tissue of Landrace pigs fed control (CG) vs. sea buckthorn pomace supplemented (FG-SBP) diet.

	CG	FG-SBP	*p*-Value (*p* < 0.05)
	LSM_SEM_ (*n* =10)	LSM_SEM_ (*n* = 10)	
	
Sum fatty acids	35.04_2.79_	37.68_2.79_	0.5118
SFA			
C12:0	0.01_0.001_	0.01_0.001_	0.8066
C14:0	0.09_0.01_	0.09_0.01_	0.7045
C15:0	0.03_0.003_	0.03_0.003_	0.5327
C16:0	5.51_0.43_	5.84_0.43_	0.5964
C18:0	6.00_0.50_	6.44_0.50_	0.5389
C20:0	0.08_0.01_	0.09_0.01_	0.3140
Sum SFA	12.41_1.00_	13.21_0.97_	0.5653
MUFA			
C16:1	0.24_0.02_	0.26_0.02_	0.5384
C18:1 *cis*-9	6.73_0.57_	7.54_0.57_	0.3253
C18:1 *cis*-11	1.70_0.14_	1.88_0.14_	0.4091
Sum MUFA	10.09_0.87_	11.29_0.87_	0.3422
PUFA			
C18:2 *n*-6	0.24_0.02_	0.25_0.02_	0.7032
C18:3 *n*-3	0.17_0.02_	0.21_0.02_	0.2637
C20:4 *n*-6	4.16_0.32_	4.38_0.32_	0.6368
C20:5 *n*-3	0.32_0.03_	0.36_0.03_	0.3234
C22:5 *n*-3	0.14_0.01_	0.13_0.01_	0.6499
C22:6 *n*-3	4.74_0.38_	4.89_0.38_	0.7726
Sum PUFA	12.24_0.95_	12.84_0.95_	0.6661
*n*-6/*n*-3 PUFA ratio	1.25_0.02_	1.27_0.02_	0.3655

For footnotes see Table 1.

**Table 5:** Feed components, chemical and fatty acid composition of control- and sea buckthorn pomace (SBP) supplemented diets [10].

	Control Group (CG)	Feeding Group (FG-SBP)
Feed components (%)		
SBP	-	12.0
Wheat	26.2	53.4
Barley	20.0	-
Triticale	20.0	-
Rye	15.0	15.0
Soybean meal	11.8	12.5
Soybean skin	2.5	-
Beet molasses	0.75	-
Dextrose	-	3.3
Nutrient composition		
Crude fat (%)	2.7	3.6
Crude protein (%)	15.4	15.6
Energy (MJ)	13.4	12.2
Lysine (%)	0.68	0.60
Fatty acid profile (%)		
C14:0	0.43	0.45
C16:0	20.5	21.5
C16:1	1.2	5.0
C18:0	2.4	2.3
C18:1*cis*-9	17.5	19.5
C18:2*n*-6	47.4	39.4
C18:3*n*-3	5.8	5.8

## Abbreviations

SBP	Sea buckthorn pomace
*n*-3 PUFA	*n*-3 polyunsaturated fatty acids
SFA	Saturated fatty acids
MUFA	Mono unsaturated fatty acids
CRH	Corticotrophin releasing hormone
MR	Mineralocorticoid receptor
GR	Glucocorticoid receptor
HPA	Hypothalamic–pituitary–adrenal
